# Interactional justice at work is related to sickness absence: a study using repeated measures in the Swedish working population

**DOI:** 10.1186/s12889-017-4899-y

**Published:** 2017-12-08

**Authors:** Constanze Leineweber, Claudia Bernhard-Oettel, Paraskevi Peristera, Constanze Eib, Anna Nyberg, Hugo Westerlund

**Affiliations:** 1 Stress Research Institute, Stockholm University, Stockholm, Sweden; 2 Department of Psychology, Stockholm University, Stockholm, Sweden; 3 Norwich Business School, University of East Anglia, Norwich, UK

**Keywords:** Interactional justice, interpersonal justice, informational justice, job insecurity, organizational justice, sickness absence, work stress, epidemiology, longitudinal studies

## Abstract

**Background:**

Research has shown that perceived unfairness contributes to higher rates of sickness absence. While shorter, but more frequent periods of sickness absence might be a possibility for the individual to get relief from high strain, long-term sickness absence might be a sign of more serious health problems. The Uncertainty Management Model suggests that justice is particularly important in times of uncertainty, e.g. perceived job insecurity. The present study investigated the association between interpersonal and informational justice at work with long and frequent sickness absence respectively, under conditions of job insecurity.

**Methods:**

Data were derived from the 2010, 2012, and 2014 biennial waves of the Swedish Longitudinal Occupational Survey of Health (SLOSH). The final analytic sample consisted of 19,493 individuals. We applied repeated measures regression analyses through generalized estimating equations (GEE), a method for longitudinal data that simultaneously analyses variables at different time points. We calculated risk of long and frequent sickness absence, respectively in relation to interpersonal and informational justice taking perceptions of job insecurity into account.

**Results:**

We found informational and interpersonal justice to be associated with risk of long and frequent sickness absence independently of job insecurity and demographic variables. Results from autoregressive GEE provided some support for a causal relationship between justice perceptions and sickness absence. Contrary to expectations, we found no interaction between justice and job insecurity.

**Conclusions:**

Our results underline the need for fair and just treatment of employees irrespective of perceived job insecurity in order to keep the workforce healthy and to minimize lost work days due to sickness absence.

**Electronic supplementary material:**

The online version of this article (10.1186/s12889-017-4899-y) contains supplementary material, which is available to authorized users.

## Background

Sickness absence is a major health concern and considered as a global measure of health status [1]. In Sweden, sickness absence has increased by 70% since 2010 and governmental spending on health insurance has increased by 11 billion Swedish crowns (around 1.2 billion Euro by December, 31 2014) between 2010 and 2014 [2].

Some important contributing factors to sickness absence are found in the work environment; such have low job control and low decision latitude at work been shown to increase the odds for sick leave [3–5]. A relatively newly established determinant of employee health is employees’ perceptions of fairness in the organization, i.e., organizational justice. Organizational justice can be divided into several sub-dimensions: the fairness of formal decision-making in the organization (procedural justice), the perceived fairness of decision outcomes (distributive justice), and the treatment of employees by supervisors (interactional justice) [6]. The interactional justice component of organizational justice is the focus of this study, and can be further divided into informational justice, i.e., receiving truthful and candid information with adequate justifications, and interpersonal justice concerning the respectful and dignified treatment from the supervisor [6].

Relatively little is known about how organizational justice relates to health and only since around the turn of the millennium research on organizational justice has been expanded to health outcomes [7]. Studies have investigated the relationship between justice perceptions and different health indicators, such as well-being, burnout, health problems, or sickness absence [8, 9]. Regarding sickness absence, the few existing studies generally suggest that perceived low organizational justice increases the risk of sickness absence. However, there is little consistency in the results and between-study differences are immense [9]. This may be due to the heterogeneity in sickness absence measures and an insufficient differentiation between long-term, short-term and frequent sickness absence. A sufficient differentiation between sickness absence measures is of importance; different mechanisms have been suggested to lie behind long and short term sick leave. While short-term sickness absence might be a means for the individual to get relief from high strain and therefore could be considered as a coping mechanism (the ‘withdrawal’ explanation), long-term sickness absence might be a sign of more serious health problems due to a prolonged stress reaction to poor work conditions (the ‘stress’ explanation) [5, 6]. However, to be able to look at different indicators of sickness absence, and to differentiate between long term and short term sickness absence, truly longitudinal studies with repeated measures of both predictor and outcome are needed. Until today, relatively few studies have examined the organizational justice–sickness absence relationship using longitudinal analysis techniques which take repeated measurements of organizational justice into account [10–12].

Consequently, the **first aim** of our study is to investigate the association between informational and interpersonal justice and long and frequent sickness absence, respectively, employing repeated measured covering a time span of six years.

The Uncertainty Management Model states that justice perceptions are of greater importance for people in insecure situations than for those in secure situations [13]. It is assumed that in uncertain situations, individuals use fairness as a heuristic to judge whether they can trust their employer and supervisor. If individuals perceive unfairness in uncertain times, they may perceive their situation as even more unpredictable, which may just increase their feelings of stress and their tendency to call in sick. Indeed, previous experimental studies indicated that affective and behavioural reactions to perceived justice were stronger among those in uncertain situations [13]. A cross-sectional study reported a stronger association between procedural injustice and stress symptoms among employees with high compared with low job insecurity [14]. Another study found that job insecurity accentuated unfairness perceptions, which related negatively to well-being, but also here evidence is cross-sectional [15]. The Uncertainty Management Model assumptions were also supported by a study which found that associations between procedural and interpersonal justice and sickness absence were dependent on experienced work-time control and perceived changes at work [16]. However, while low interpersonal justice may increase the risks for long and frequent sickness absence due to high stress levels, low informational justice may lead to decreases in frequent but short absence when there is high job insecurity, since employees may go to work and seek information to reduce their uncertainty. Still, to the best of our knowledge, very little is known about the role of informational and interpersonal justice for sickness absence in times of high uncertainty at work, such as high job insecurity.

Thus, our **second aim** to investigate the association between interpersonal and informational justice at work and long and frequent sickness absence under conditions of job insecurity.

## Methods

### Study population

**Fig. 1:**
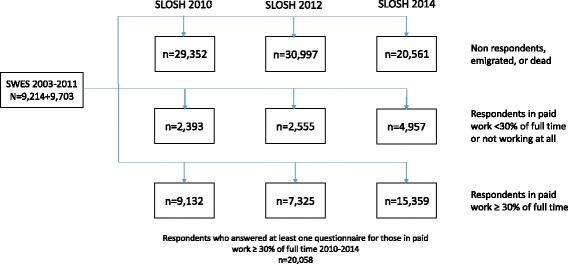
Flow chart illustrating the number of participants in the study

The study population consisted of the participants of SLOSH (Swedish Longitudinal Occupational Survey of Health), a longitudinal cohort study with a focus on the association between work organization, work environment, and health (www.slosh.se). SLOSH follows participants of the Swedish Work Environment Surveys (SWES) 2003-2011 (n=40,877). SWES is conducted biennially by Statistics Sweden and consist of a subsample of gainfully employed people aged 16-64 from the Labour Force Survey (LFS) representative of the Swedish working population. Since the start of SLOSH in 2006, eligible SWES participants were invited every second year to respond to a postal questionnaire in two versions, one for those currently in paid work and one for those permanently or temporarily outside the labour force. Items of interest for the present study were measured since 2010. The present paper is based on participants who responded at least once on the questionnaires for those in paid work between the 2010 (wave 3) and the 2014 (wave 5) data collection (Fig. 1). Response rates were 57% in 2010 (n=11,525), 57% in 2012 (n=9,880), and 53% in 2014 (n=20,316). After exclusion of self-employed and farmers the final study sample consisted of 19,493 individuals with 58,479 observations. All participants gave their informed consent. Both SLOSH and the present study have been approved by the Regional Research Ethics Board in Stockholm.

### Main measurements

#### Interactional organizational justice

Interactional justice was measured by items from the Stress profile – managerial leadership, [17] a measure originally constructed to measure leadership climate. In a first step, we identified seven out of nine items^1^ which we regarded as comparable to items used in scales to measure interactional justice [6, 18, 19]. All items were responded to on a four-point Likert-scale ranging from 1='yes, often’ to 4='no, never', thus higher values indicate lower justice. An exploratory factor analysis based on a random half of the 2010 data suggested a two-factor solution; one factor with four items about the supervisor’s respect and dignity in the treatment of the employees, and one with three items about information and expectations. Following Colquitt, [6] we call these factors ‘interpersonal’ and ‘informational’ justice. A subsequent confirmatory factor analysis based on the other random 50% of the sample provided satisfying fit values. The two-factor model with correlated latent variables (r=.84) provided better fit than the one factor model or a two-factor model with uncorrelated latent variables. Item wordings for each factor and model fit are presented in Additional file 1. Factor analyses were conducted in Mplus7.4.

To calculate scale values for interpersonal and informational justice, we subtracted the number one from the original value (to get a range from 0 to 3) and calculated the mean of the four and three items, respectively. Cronbach’s alpha varied over the waves between 0.89 and 0.90 for interpersonal justice, and between 0.80 and 0.81 for informational justice, indicating a high interrelation between justice items.

#### Job insecurity

Job insecurity was measured with three items about worries losing one’s job [20]. Response options ranged from 1=totally disagree to 5=totally agree. After subtracting one from the original value, we calculated the mean of the three items reflecting job insecurity for each wave. Higher values indicated more job insecurity. Cronbach’s alpha was 0.95 in all waves, indicating a high interrelation between justice items.

#### Sickness absence

Sickness absence (excluding leave due to care of sick children) was measured in two ways [21, 22]. *Long-term sickness absence* was measured by a question regarding days of sickness absence over the past year. In line with definitions used in earlier studies, [23, 24] participants were defined as having been on long sickness absence if they stated that they had taken sick leave 31 days or more during the past 12 months. *Frequent sickness absence* was measured by a question covering number of occasions of sickness absence for up to one week during the past year. Participants were defined as having been on frequent sickness absence if they responded that they had taken sick leave twice or more during the past 12 months.

#### Covariates

Age, sex, socio-economic position and marital status were included as covariates; these variables are likely to influence both the outcome and the exposure [25, 26]. All covariates except marital status were obtained from register data linked to questionnaire responses by means of the unique Swedish ten-digit personal identification number. Age was assessed as the participants’ age at the end of the year the questionnaire was answered. A measure for sex was dichotomous. The measure of socio-economic position was based on the Swedish socio-economic classification with original 18 basic categories and dichotomised into manual and non-manual employees [27]. Marital status was obtained by a single question with response alternatives ‘single’ or ‘married/cohabiting’.

### Data analysis

Data were analysed using generalized estimating equations (GEE), a method for longitudinal data simultaneously analysing variables at different time points by assuming a priori a certain working correlation structure for the repeated measurements of the outcome variable [28]. GEE analyses were carried out in Proc Genmod in the statistical package SAS (version 9.4). Log-linked binominal regression models were specified to calculate risk ratios. The analyses in the GEE models were performed using an unstructured working correlation structure, a form that is most efficient and useful when there is data from relatively few time points [29]. We analysed the relationship between justice perceptions and long and frequent sickness absence, respectively. Separate models were run for interpersonal and informational justice. Measures of interactional justice and job insecurity were grand-mean centered. First, all study variables were analysed separately in their relation to long and frequent sickness absence, respectively (Model 0). Secondly, to answer the question whether lower levels of justice at work and job insecurity were associated with sickness absence we included justice, job insecurity, and time measured as a continuous variable simultaneously (Model 1). In the next step we added an interaction term between justice and job insecurity, thus testing if lower levels of justice are more harmful under conditions of higher job insecurity (Model 2), and in a last model (Model 3), we adjusted for demographical factors (i.e., age, sex, socio-economic position, marital status). All variables were used as time-varying covariates.

As the standard GEE pool together between participants (cross-sectional) and within participants (longitudinal) relationships, we additionally applied autoregressive GEE so as to remove the between-subjects part of the relationship between informational and interpersonal justice, respectively, and sickness absence [30]. In autoregressive GEE the value of the outcome variable at a specific time point is predicted by the very same variable's value of the previous time point t-1 (autoregression) and the value of the predictor variable at the previous time point t-1 [31]. For details, see Additional file 2.

To test if missing data were “informative” or “ignorable” [30], we divided the population into two groups, one including the subjects without any missing data over the study period and one including the subjects with missing data at one or more of the repeated measurements. The two groups were then compared to each other regarding the values of the outcome variables and the covariates at the first measurement. Results are presented in Additional file 3.

## Results

Descriptive information is presented in Table 1. At baseline, 5% of the study population reported having been on long sickness absence during the past 12 months and 20% reported having been on frequent sickness absence. Job insecurity (range 0-4) was generally rated as low with a mean of 0.47 (SD=0.92). All potential confounders were independently associated with long and frequent sickness absence, respectively (see Additional file 4).

**Table 1:** Characteristics of the study participants at wave 1 (2010) – wave 3 (2014)

Variable	Wave 1	Wave 2	Wave 3
Sex, n (%)			
Male	3,882 (42.6)	3,276 (42.3)	6,937 (42.5)
Female	5,235 (57.4)	4,475 (57.7)	9,396 (57.5)
Age^a^, mean (SD)	49.2 (10.4)	51.3 (10.4)	51.70 (10.5)
SES, n (%)			
Manual	2,860 (32.4)	2,404 (31.8)	4,821 (30.3)
Non-manual	5,966 (67.6)	5,148 (68.2)	11,075 (69.7)
Marital status, n (%)			
Married, cohabiting	7,093 (79.5)	6,094 (79.3)	12,818 (79.2)
Single	1,830 (20.5)	1,589 (20.7)	3,366 (20.8)
Interpersonal justice^b^, mean (SD)	1.13 (0.80)	1.12 (0.77)	1.00 (0.76)
Informational justice^b^, mean (SD)	0.78 (0.67)	0.79 (0.66)	0.70 (0.63)
Job insecurity^c^, mean (SD)	0.47 (0.92)	0.42 (0.88)	0.36 (0.81)
Long sickness absence, n (%)			
0-30 days during the past 12 months	8,524 (94.7)	7,030 (92.1)	15,217 (94.1)
31 days or more during the past 12 months	478 (5.3)	599 (7.8)	960 (5.9)
Frequency of sickness absence, n (%)			
< 2 periods during the past 12 months	6,823 (80.1)	5,384 (79.7)	11,890 (80.2)
2 periods or more during the past 12 months	1,699 (19.9)	1,370 (20.3)	2,933 (19.8)

^a^ range: 20-72

^b^ range: 0-3 (higher values indicate lower levels of perceived justice)

^c^ range: 0-4

### Interpersonal justice

Tables 2 and 3 show the estimated risk ratios between interpersonal justice and long (Table 2) and frequent (Table 3) sickness absence. In the crude models (Model 0) lower levels of interpersonal justice were statistically significantly related to an increased risk of long (RR=1.19, 95% CI: 1.11-1.27) as well as frequent sickness absence (RR=1.14; 95% CI: 1.10-1.18). These relations did only marginally change when controlling for higher job insecurity and time. Also in the autoregressive models (Model 1), lower levels of interpersonal justice were statistically significantly related to both long (RR=1.10; 95% CI: 1.00-1.20) and frequent (RR=1.05; 95% CI: 1.00-1.10) sickness absence.

**Table 2:** Results of the generalized estimating equations (GEE) analyses of the association between interpersonal justice (higher values indicate lower levels of perceived justice) and long sickness absence (31 days or more the previous 12 months), presented as Risk ratios (RR) with 95% Cis

	Model 0		Model 1		Model 2		Model 3	
Results for standard GEE	RR	95% CI	RR	95% CI	RR	95% CI	RR	95% CI
Interpersonal justice	1.19	1.11-1.27	1.17	1.09-1.25	1.16	1.09-1.25	1.18	1.10-1.28
Job insecurity	1.13	1.07-1.19	1.11	1.05-1.17	1.09	1.03-1.16	1.12	1.04-1.20
Justice*insecurity					1.04	0.98-1.10	1.03	0.96-1.10
Results for autoregressive GEE								
Interpersonal justice			1.10	1.00-1.20	1.10	1.00-1.20	1.10	1.01-1.21
Job insecurity			1.08	1.01-1.16	1.09	1.01-1.17	1.11	1.03-1.19
Previous sickness absence			3.44	2.81-4.22	3.44	2.81-4.22	2.73	2.20-3.39
Justice*insecurity					0.99	0.91-1.08	0.98	0.90-1.07

Model 1: interpersonal justice, job insecurity, and time included contemporarily; Model 2: Model 1 plus interaction term included; Model 3: Model 2 plus age, sex, socio-economic position, and marital status

**Table 3:** Results of the generalized estimating equations (GEE) analyses of the association between interpersonal justice (higher values indicate lower levels of perceived justice) and frequent sickness absence (two periods or more during the previous 12 months), presented as risk ratios (RR) with 95% CIs

	Model 0		Model 1		Model 2		Model 3	
Results for standard GEE	RR	95% CI	RR	95% CI	RR	95% CI	RR	95% CI
Interpersonal justice	1.14	1.10-1.18	1.13	1.09-1.16	1.12	1.09-1.16	1.14	1.10-1.12
Job insecurity	1.10	1.08-1.13	1.07	1.05-1.10	1.07	1.04-1.10	1.05	1.01-1.08
Justice*insecurity					1.00	0.88-1.13	1.01	0.98-1.04
Results for autoregressive GEE								
Interpersonal justice			1.05	1.00-1.10	1.05	1.00-1.10	1.05	1.00-1.11
Job insecurity			1.08	1.04-1.12	1.08	1.04-1.12	1.07	1.03-1.11
Previous sickness absence			4.56	4.17-5.00	4.56	4.17-5.00	4.18	3.80-4.59
Justice*insecurity					0.99	0.95-1.04	0.98	0.02-1.03

Model 1: interpersonal justice, job insecurity, and time included contemporarily; Model 2: Model 1 plus interaction term included; Model 3: Model 2 plus age, sex, socio-economic position, and marital status

We then investigated the relation of interpersonal justice to sickness absence in interaction with job insecurity (Model 2). We found a statistically significant relationship between lower levels of interpersonal justice and long (RR=1.16; 95% CI: 1.09-1.25) and frequent (RR=1.12; 95% CI: 1.09-1.16) sickness absence. However, the interaction terms did not reach statistical significance. Also here, results for the autoregressive relationship reached statistical significance, both in regard to long (RR=1.10; 95% CI: 1.00-1.20) and frequent sickness absence (RR=1.05; 95% CI: 1.00-1.10). Additional control for demographic confounders (Model 3) altered results only marginally.

Higher levels of job insecurity were statistically significantly related to long as well as frequent sickness absence in all models.

### Informational justice

Tables 4 and 5 present the estimated risk ratios between informational justice and long (Table 4) and frequent (Table 5) sickness absence. Not controlling for any confounder (Model 0), lower levels of informational justice were statistically significantly related to an increased risk of both long (RR=1.14; 95% CI: 1.06-1.23) and frequent (RR=1.16; 95% CI: 1.12-1.20) sickness absence. These relationships altered only marginally when controlling for job insecurity (Model 1). In the autoregressive analyses, lower levels of informational justice were no longer associated with an increased risk of long sickness absence (RR=1.02; 95% CI: 0.92-1.13). Still, lower levels of informational justice were significantly associated to frequent sickness absence, even when controlling for previous episodes of frequent sickness absence (RR=1.08; 95% CI: 1.02-1.13).

**Table 4:** Results of the generalized estimating equations (GEE) analyses of the association between informational justice (higher values indicate lower levels of perceived justice) and long sickness absence (31 days or more the previous 12 months), presented as risk ratios (RR) with 95% CIs

	Model 0		Model 1		Model 2		Model 3	
Results for standard GEE	RR	95% CI	RR	95% CI	RR	95% CI	RR	95% CI
Informational justice	1.14	1.06-1.23	1.12	1.03-1.21	1.12	1.03-1.21	1.21	1.10-1.33
Job insecurity	1.13	1.07-1.19	1.12	1.06-1.18	1.12	1.06-1.19	1.14	1.07-1.23
Justice*insecurity					1.00	0.93-1.07	0.98	0.91-1.06
Results for autoregressive GEE								
Informational justice			1.02	0.92-1.13	1.02	0.92-1.13	1.09	0.99-1.21
Job insecurity			1.08	1.00-1.15	1.08	1.00-1.16	1.11	1.03-1.19
Previous sickness absence			3.48	2.88-4.19	3.48	1.89-4.19	2.83	2.33-3.43
Justice*insecurity					0.98	0.90-1.07	0.96	0.88-1.04

Model 1: informational justice, job insecurity, and time included contemporarily; Model 2: Model 1 plus interaction term included; Model 3: Model 2 plus age, sex, socio-economic position, and marital status

**Table 5:** Results of the generalized estimating equations (GEE) analyses of the association between informational justice (higher values indicate lower levels of perceived justice) and frequent sickness absence (2 periods or more during the previous 12 months), presented as risk ratios (RR) with 95% CIs

	Model 0		Model 1		Model 2		Model 3	
Results for standard GEE	RR	95% CI	RR	95% CI	RR	95% CI	RR	95% CI
Informational justice	1.16	1.12-1.20	1.14	1.10-1.19	1.15	1.11-1.19	1.16	1.11-1.21
Job insecurity	1.10	1.08-1.13	1.07	1.05-1.10	1.08	1.05-1.11	1.06	1.02-1.09
Justice*insecurity					0.98	0.95-1.01	0.98	0.95-1.01
Results for autoregressive GEE								
Informational justice			1.08	1.02-1.13	1.07	1.01-1.13	1.08	1.02-1.14
Job insecurity			1.06	1.02-1.10	1.05	1.01-1.09	1.05	1.00-1.09
Previous sickness absence			4.60	4.23-5.01	4.61	4.23-5.02	4.20	3.84-4.60
Justice*insecurity					1.02	0.97-1.07	1.00	0.94-1.06

Model 1: informational justice, job insecurity, and time included contemporarily; Model 2: Model 1 plus interaction term included; Model 3: Model 2 plus age, sex, socio-economic position, and marital status

Investigating the relationship between informational justice and sickness absence in interaction with job insecurity (Model 2), we found lower levels of informational justice to be associated with an increased risk of long (RR=1.12; 95% CI: 1.03-1.21) and frequent (RR=1.15; 95% CI: 1.11-1.19) sickness absence. When controlling for demographic confounders the relationship with long sickness absence was slightly strengthened (RR=1.21; 95% CI: 1.10-1.33), but remained unchanged for frequent sickness absence (RR=1.16; 95% CI: 1.11-1.21). Turning to the autoregressive models, we found that lower levels of informational justice were not significantly related to long sickness absence (RR=1.02; 95% CI: 0.92-1.13), but did relate to an increased risk of frequent sickness absence (RR=1.07; 95% CI: 1.01-1.13). These results did not change substantially when controlling for demographic confounders (Model 3). The interaction term between informational justice and job insecurity did not reach statistical significance in any of the models.

Higher levels of job insecurity were related to both long as well as frequent sickness absence in all models including informational justice, although risk ratios were mostly low.

## Discussion

In line with our expectations, perceptions of lower levels of interpersonal and informational justice increased the risk of long as well as frequent sickness absence, using standard GEE. These findings suggest that lower levels of justice at work might relate both to increased withdrawal behaviour in form of shorter, but more frequent sickness absence periods and to more serious health effects as indicated by an increased risk of longer sickness absence episodes. Thus, our results are in line with earlier research showing that low levels of organizational justice are associated with an increased risk for ill-health and sickness absence [16, 32, 33]. However, observed differences in risk ratios regarding the two different sickness absence measures were small and future studies should further investigate possible differentiating effects of organizational justice on long and frequent sickness absence.

Based on the results from the standard GEE, no conclusions can be drawn regarding the temporal relationship between low justice perceptions and sickness absence, i.e. we do not know if effects of organizational justice are due to differences in perceived organizational justice between participants (cross-sectional) or within participants (longitudinal). Thus, using autoregressive GEE we removed the between-subjects part of the relationships by taking previous periods of sickness absence into account and investigated possible longitudinal associations between organizational justice and sickness absence. Regarding interpersonal justice, autoregressive GEE revealed that the increased risks of long and frequent sickness absence, respectively, can be explained by within-effects, i.e., having experienced a decrease of interpersonal justice at a personal level related to an increase in sickness absence in that person. Regarding informational justice, we found no significant increased risk of long sickness absence using autoregressive GEE, i.e., our results indicate that our findings from standard GEE rely mainly on between-effects. In other words, people with lower justice perceptions have an increased risk of long sickness absence in comparison to those with higher justice perceptions. With regard to frequent sickness absence, we found a small statistically significant effect of informational justice, indicating that changes in informational justice perceptions were related to increased withdrawal behaviour in form of frequent sickness absence episodes.

In a next step, we investigated the relations justice and sickness absence in interaction with job insecurity. In contrast to our expectations, we could not find any statistically significant interaction between justice perceptions and job insecurity; i.e., the association between lower levels of justice perceptions and any type of sickness absence did not differ under conditions of higher levels of job insecurity. It is quite possible that this null-finding can be explained by the general low levels of perceived job insecurity in our study population; seventy percent of the study population did not report any job insecurity at all.

Our results contradict previous findings showing that the relationship between low justice and strain was moderated by uncertainty-related aspects of the workplace [14, 16]. However, while we measured job insecurity in terms of being afraid of losing one’s job, Elovainio et al. operationalised insecurity in terms of control over working hours and negative changes [16]. Also, the authors of the uncertainty management model are quite ambiguous in their definitions of uncertainty and it might make a difference whether we investigate environmental uncertainty (e.g., job insecurity) or personal uncertainty (e.g., lack of standing/respect/belonging to a work group, self-esteem issues). Also, job insecurity might not be bad for everyone, e.g., a temporary work contract might be accepted as a step in career or as a possibility which offers flexibility.

### Strengths and limitations

Strengths of this study are the large study population approximately representative of the Swedish working population covering a wide range of occupations and sectors, and its longitudinal design. Furthermore, our analysis relies on a longitudinal analysis technique, which allows an efficient adjustment for correlated data and provides more robust results than traditional regression analysis. Also, GEE provides consistent estimates even with miss-specified correlation structures which ensures the robustness of the results. Despite these strengths, there are also limitations to consider. We based our analyses on a sample where some individuals had only responded to one or two of the three included waves. One problem that may arise from missing data and drop out is that the distribution of the observed data may not be the same as the distribution of the complete data. The GEE models may yield biased estimates unless drop out is MCAR (missing completely at random) although it is not clear from the literature how important this bias really is [30]. However, a sensitivity analysis based on a sample including only those who had answered on at least two out of three waves provided very similar results as the here presented ones. Thus, there is some evidence that missing values are not a problem for our analysis. Another limitation might be that all variables are based on self-reports, which could inflate the risk for common method bias. It has, however, been shown that same-method observed score correlations are relatively accurate representations of their true-score counterparts and that in many cases, common method bias may be trivially small [34, 35]. Also, estimates may suffer from the omitted-variable bias, such as e.g. personality traits might correlated with measures that are used in the analyses [36]. Another issue that is of major importance for the size of estimated effects is the time frame. Whether or not the time frame of two years is optimal is unknown and a shorter time span might have been better suited to catch withdrawal reactions. Another possible shortcoming is that our scale measuring certain dimensions of justice was originally developed to measure leadership climate. Still, our items are comparable to items used in other justice scales and show good internal reliability. A further shortcoming is found in the fact that heterogeneity was not taken into account and further studies should investigate potential effects of gender as there are indications that women reacted more strongly with ill-health to injustice perceptions compared to men [37, 38]. Further, employees are not randomly assigned into workplaces and failure to account for sorting of employees could bias estimated effects [36]. We addressed this concern at least partly by using autoregressive GEE, controlling for prior health. However, despite using autoregressive GEE in addition to standard GEE, causality cannot be established and reversed effects as suggested by Lang et al. and Ybema et al. cannot be excluded [10, 39]. Also, our outcomes of sickness absence were based on self-report data and sickness absence gathered from population registries or company records would have been preferable [40–42]. However, several European studies report a relatively good agreement between self-reported and recorded days of sickness [43, 44]. Thus, taken together our results indicate that actions to improve job security and justice at work might be well suited to decrease sickness absence rates.

## Conclusions

Our findings indicate that lower levels of informational and interpersonal justice are associated with an increased risk for long and frequent sickness absence. Also, higher levels of job insecurity turned out as an important predictor of both long and frequent sickness absence. Perceived fairness at work is a modifiable aspect of the work environment, as is job insecurity. Organizations have significant control over both aspects and our results suggest that organizations may gain by investing or improving their policies and rules for fair treatment of their workforce and by improving job security. Further, organization might gain by selection of supervisors for their qualities associated with fair practices, training supervisors in justice principles, and implement performance management practices for them that consider their use of organizational justice [45]. Indeed, training in justice principles has been shown to be successful in different organizational contexts and is connected with relative lower costs as compared to other justice interventions (e.g. raising pay) [46].

## Additional files

Additional file 1: Model fits for interactional justice and factor loadings. (DOCX 27 kb)

Additional file 2: Standard and autoregressive GEE models. (DOCX 25 kb)

Additional file 3: Attrition analysis comparing those who had full information in all waves compared to those with missing information on at least one variable at least at one wave. (DOCX 27 kb)

Additional file 4: Results of standard generalized estimating equations (GEE) analyses of the association between covariates and long and frequent sickness absence, respectively, presented as risk ratios (RR) with 95% CIs. RRs represent the uncontrolled risk ratios. (DOCX 26 kb)

## Abbreviations

CI: Confidence interval
GEE: Generalised estimating equations
LFS: Labour Force Survey
RR: Risk ratio
SD: Standard deviation
SWES: Swedish Work Environment Survey

## Footnotes

1 One of the original 10 items in the stress profile (“I receive criticism from my boss if I have done something that is not good”) was not taken into consideration as it was not measured in all waves.
