# Supplementary material for: Interactional justice at work is related to sickness absence: a study using repeated measures in the Swedish working population
Source: BMC Public Health. 2017 Dec 8;17:912. doi: 10.1186/s12889-017-4899-y (PMC5721595; doi:10.1186/s12889-017-4899-y)
Supplement: Supplementary file 1 — Model fits for interactional justice and factor loadings. (DOCX 27 kb) [file 12889_2017_4899_MOESM1_ESM.docx]

**Additional file 1.** Model fits for interactional justice and factor loadings

|  | **χ2 (DF)** | **p** | **RMSEA** | **CFI** | **SRMR** |
| --- | --- | --- | --- | --- | --- |
| Null factor model | 16832.923 (21) | 0.0000 | 0.443 | 0.000 | 0.453 |
| One factor model | 1063.454 (14) | 0.0000 | 0.136 | 0.938 | 0.043 |
| Two factor, uncorrelated | 3241.816 (14) | 0.0000 | 0.238 | 0.808 | 0.310 |
| Two factor, correlated | 338.391 (13) | 0.0000 | 0.078 | 0.981 | 0.020 |
|  |  |  |  |  |  |
| **Model: Two factor, correlated** | | | | Factor loading | |
| Item | | | | **F1** | **F2** |
| I receive praise from my boss if I have done something good | | | | 0.814 |  |
| My boss shows that he/she cares how things are for me and how I feel | | | | 0.839 |  |
| My boss encourages my participation in the scheduling of my work | | | | 0.826 |  |
| My boss takes the time to become involved in his/her employees’ professional development | | | | 0.834 |  |
| My boss gives me the information I need. | | | |  | 0.736 |
| I have a clear picture of what my boss expects of me | | | |  | 0.725 |
| My boss explains goals and sub-goals for our work so that I understand what they mean for my particular part of the work | | | |  | 0.822 |
