# Supplementary material for: Interactional justice at work is related to sickness absence: a study using repeated measures in the Swedish working population
Source: BMC Public Health. 2017 Dec 8;17:912. doi: 10.1186/s12889-017-4899-y (PMC5721595; doi:10.1186/s12889-017-4899-y)
Supplement: Supplementary file 2 — Standard and autoregressive GEE models. (DOCX 25 kb) [file 12889_2017_4899_MOESM2_ESM.docx]

**Additional file 2.**

1. Standard GEE model on interpersonal justice and risk of long sickness absence –based on number of observations from individuals

$$\left( \begin{matrix} Long sickness absence 2010 \\ Long sickness absence 2012 \\ Long sickness absence 2014 \end{matrix} \right)=\beta_{0}+\beta_{1}\left( \begin{matrix} Interpersonal Justice 2010 \\ Interpersonal Justice 2012 \\ Interpersonal Justice 2014 \end{matrix} \right)$$

1. Autoregressive GEE model on prior interpersonal injustice and risk of long sickness absence – based on number of observations from individuals (The autoregressive models are based on fewer number of observations because the data is restructured and the first time point disregarded in the analyses)

$$\left( \begin{matrix} Long sickness absence 2012 \\ Long sickness absence 2014 \end{matrix} \right)=\beta_{0}+\beta_{1}\left( \begin{matrix} Interpersonal Justice 2010 \\ Interpersonal Justice 2012 \end{matrix} \right)+\beta_{2}\left( \begin{matrix} Long sickness absence 2010 \\ Long sickness absence 2012 \end{matrix} \right)$$
