# Supplementary material for: Interactional justice at work is related to sickness absence: a study using repeated measures in the Swedish working population
Source: BMC Public Health. 2017 Dec 8;17:912. doi: 10.1186/s12889-017-4899-y (PMC5721595; doi:10.1186/s12889-017-4899-y)
Supplement: Supplementary file 3 — Attrition analysis comparing those who had full information in all waves compared to those with missing information on at least one variable at least at one wave. (DOCX 27 kb) [file 12889_2017_4899_MOESM3_ESM.docx]

|  |  | |  |
| --- | --- | --- | --- |
|  | Part of sample with full information  n=2492 | Part of sample including with some missing data  n=16973 |  |
| Measured at baseline (2010) |  |  | p |
|  | mean±SD | mean±SD |  |
| Age mean | 48.77±8.48 | 49.42±11.03 | <.01 |
| Interpersonal justice | 1.11±0.77 | 1.14±0.80 | n.s. |
| Informational justice | 0.77±0.66 | 0.78±0.68 | n.s. |
| Job insecurity | 0.44±0.87 | 0.48±0.95 | <.05 |
|  |  |  |  |
|  | **n (%)** | **n (%)** |  |
| Women | 1455 (58.83) | 3769 (56.89) | n.s. |
| Non-manual worker | 3427 (69.54) | 2539 (65.14) | <.0001 |
| Married or cohabiting | 3949 (79.3) | 3144 (79.74) | n.s. |
| Long sickness absence | 98 (3.93) | 380 (5.84) | <.001 |
| Frequent sickness absence | 477 (19.14) | 1222 (20.27) | n.s. |

**Additional file 3.** Attrition analysis comparing those who had full information in all waves compared to those with missing information on at least one variable at least at one wave.
