# Supplementary material for: Interactional justice at work is related to sickness absence: a study using repeated measures in the Swedish working population
Source: BMC Public Health. 2017 Dec 8;17:912. doi: 10.1186/s12889-017-4899-y (PMC5721595; doi:10.1186/s12889-017-4899-y)
Supplement: Supplementary file 4 — Results of standard generalized estimating equations (GEE) analyses of the association between covariates and long and frequent sickness absence, respectively, presented as risk ratios (RR) with 95% CIs. RRs represent the uncontrolled risk ratios. (DOCX 26 kb) [file 12889_2017_4899_MOESM4_ESM.docx]

**Additional file 4**. Results of standard generalized estimating equations (GEE) analyses of the association between covariates and long and frequent sickness absence, respectively, presented as risk ratios (RR) with 95% CIs. RRs represent the uncontrolled risk ratios.

|  | **Long sickness absence** | | **Frequent sickness absence** | |
| --- | --- | --- | --- | --- |
|  | **RR** | **95% CI** | **RR** | **95% CI** |
| Age | 1.01 | 1.01-1.02 | 0.98 | 0.98-0.98 |
| Sex (female) | 1.94 | 1.76-2.15 | 1.61 | 1.52-1.71 |
| Non-manual position | 0.66 | 0.60-0.72 | 0.83 | 0.78-0.88 |
| Married | 0.80 | 0.72-0.89 | 0.86 | 0.81-0.91 |
